# Advancing Sustainable Production of High-Performance Cellulose Pulps

**DOI:** 10.3390/ma18214968

**Published:** 2025-10-30

**Authors:** María Guadalupe Morán-Aguilar, Iván Costa-Trigo, Gabriela A. Bastida, André Mazega, Josep Duran, José Manuel Domínguez, Fabiola Vilaseca

**Affiliations:** 1 Advanced Biomaterials and Nanotechnology (BIMATEC), Department of Chemical and Agricultural Engineering, and Agrifood Technology, High Polytechnic School, University of Girona, C/Maria Aurèlia Capmany, 61, 17003 Girona, Spain; josep.duran@udg.edu; 2 LNEG—National Laboratory of Energy and Geology, Bioenergy and Biorefineries Unit, Estrada do Paço do Lumiar, 22, 1649-038 Lisbon, Portugal; ivan.trigo@lneg.pt; 3 LEPAMAP-PRODIS Research Group, Department of Chemical and Agricultural Engineering, and Agrifood Technology, High Polytechnic School, University of Girona, C/Maria Aurèlia Capmany, 61, 17003 Girona, Spain; gabriela.bastida@udg.edu (G.A.B.); 4 Industrial Biotechnology and Environmental Engineering Group “BiotecnIA”, Chemical Engineering Department, Faculty of Sciences, University of Vigo (Campus Ourense), 32004 Ourense, Spain; jmanuel@uvigo.es

**Keywords:** enzymatic hydrolysis, cellulose fibrillation, sulphite pulp, eucalyptus pulp, thermomechanical pulp, high-performance

## Abstract

**Highlights:**

**What are the main findings?**

- Enzymatic hydrolysis pretreatment of industrial pulps
- Pulp composition influencing the enzymatic performance
- Enhanced conditions for high-performance cellulose pulps

**What is the implication of the main finding?**

- Sustainable methodology to produce cellulose pulps
- Lower environmental impact and alignment with circular economic principles
- Improvements in tensile strength, air permeability, hydrophobicity, and internal bonding

**Abstract:**

With a growing demand for renewable resources in high-performance materials, sustainable methods are preferred for their lower environmental impact and alignment with circular economy principles. Among these, enzymatic hydrolysis remains relatively underexplored yet shows strong potential for cellulose fibrillation, offering a promising route that may lower energy requirements by minimizing the need for extensive refining compared to conventional mechanical or chemical approaches. In this study, enzyme cocktails rich in cellulase and xylanase were applied to three industrial pulps, sulphite, bleached Kraft eucalyptus and thermomechanical pine, to produce high-performance cellulose pulps. Treatments were carried out using varying enzyme loads (5–40 filter paper units per gram of dry pulp, FPU/gdp) and reaction times (1–16 h). The resulting chemical composition, structural morphology, and physical–mechanical properties were systematically evaluated. The findings revealed that pulp composition strongly influenced enzymatic treatment, affecting surface fibrillation, fibre aggregation, swelling, and fibre shortening. Under optimized conditions, enzymatic pretreatment significantly enhanced paper performance, with improvements in tensile strength, air permeability, hydrophobicity, and internal bonding. Overall, enzymatic hydrolysis represents a sustainable solution and a strategy which could reduce energy expenditures to high-performance cellulose pulps, suitable as reinforcing fibres in packaging applications.

## 1. Introduction

Pulp and paper products are considered one of the most important industrial materials as they are closely related to economic and social development [1]. In this sense, an annual production of paper and fibreboard of 4197 MT (Million Tonnes) has been reported, which represents significant resource consumption and considerable environmental impact [2].

Papermaking is a complex process aimed at producing paper sheets with maximum strength at the lowest cost from fibres of different origins [3]. Likewise, it has been proved that the pulp and papermaking industry requires large amounts of water, chemicals and energy [4]. For instance, the production of 1 ton of paper using a traditional process generates approximately 950 kg of CO_2_ greenhouse gas emissions, which represents about 6% of the world’s final industrial energy consumption and 9% of the greenhouse gas emissions of the manufacturing sector [5]. The refining process is a crucial and mandatory step to produce paper with adequate strength properties; otherwise, bleached Kraft pulp does not provide sufficient bonding strength. Consequently, mechanical treatment (refining) is used to increase fibre bonding and develop the resistance of the paper [3]. However, it is important to note that this stage represents almost 30% of the total electrical energy consumed in the papermaking process [2,6]. Therefore, considering the annual paper production, minor modifications in the processing could help to reduce energy consumption and, consequently, CO_2_ emissions on a global scale.

Moreover, cellulosic raw materials can also be used as a resource to produce renewable alternatives to synthetic resins such as urea, phenol, and melamine– formaldehyde, which represent about 70% of the global demand in engineered wood. Formaldehyde is classified as a human carcinogen by the World Health Organization (OMS) and the International Agency for Research on Cancer (IARC). In this regard, serious environmental and health concerns have been raised [7,8].

Consequently, there is increasing scientific, environmental, industrial, and healthcare interest in advancing cost-effective technologies that harness natural resources to generate cellulosic-based fibres and resins, aimed at creating materials that are both more flexible and more durable [9].

In this context, employing enzymes as green catalysts in the cellulose industry offers a sustainable, energy-efficient alternative for producing materials with enhanced mechanical properties, while simultaneously reducing refining time as well as reliance on chemicals and energy. Moreover, developing a renewable cellulose-based resin could serve as a sustainable alternative to conventional chemical products [1]. Enzyme-catalysed methods offer distinct advantages, including the absence of hazardous residue formation, operation under mild conditions, and the potential for enzyme recovery through immobilisation, thereby supporting the development of zero-waste processes [10,11,12].

However, obtaining cellulose derivatives or their modifications (nanofibres, nanorods and hybrids) has mainly focused on the study and application of cellobiohydrolases and endoglucanases as catalysts in the cellulose hydrolysis process [10,13,14] which may be insufficient for effective cellulose modification even when a pretreatment step (defibrillation) is employed to increase enzymatic accessibility [15,16]. Nevertheless, recent research demonstrated xylanase contribution as accessory enzymes for the generation of more efficient processes (less enzyme dosage and reduced reaction times), and to the production of materials with enhanced chemical, physical, and mechanical characteristics [17].

Xylanases are enzymes that catalyse the hydrolysis of residual hemicellulose, which acts as a barrier to cellulolytic enzymes in lignocellulosic materials. Furthermore, they act synergistically with cellulolytic enzymes by promoting the amorphization of cellulose fibres and increasing the access of cellulases to cellulose [18]. Although, to our knowledge, not many studies have addressed the importance of using complex cocktail enzymes during the enzymatic hydrolysis of cellulose to obtain cellulose derivatives or cellulosic materials with better characteristics (high aspect ratio, crystallinity, and chemical composition) [19].

The production of bleached pulps with adequate mechanical properties for the paper industry involves the intensive use of chemicals and additional stages that demand high energy consumption. In this context, the comparative study of the chemical composition of different industrial pulps may represent a turning point for understanding the enzymatic behaviour of cellulases and xylanases. Such knowledge would enable the development of fibres with improved chemical, physical, and mechanical properties through a single enzymatic treatment step, based on a sustainable technology that could further be optimised within a circular process framework.

Building on this rationale, this study examines the effects of enzymatic loading and reaction time using a multienzyme cocktail (mainly cellulases and xylanases) on three industrial pulps sulphite, eucalyptus Kraft, and thermomechanical. The effects on chemical composition, as well as on physical and mechanical properties, were evaluated to design cellulosic materials with enhanced performance, suitable for sustainable reinforcement in packaging applications.

## 2. Materials and Methods

### 2.1. Raw Materials

Sulphite pulp from spruce was supplied by Domsjö (Örnsköldsvik, Sweden). Bleached kraft eucalyptus pulp was supplied by ENCE Energía & Celulosa from Navia (Spain). Thermomechanical pulp from pine, produced from pre-steamed wood chips refined through multiple cycles at high temperature and pressure, was provided by Mas Clarà S.A (Girona, Spain).

### 2.2. Reagents

The commercial enzyme Cellic CTec2 (Cellic CTec2-SAE0020) was obtained from Sigma-Aldrich (St. Louis, MO, USA) and used for fibre pretreatment. The enzymatic activity quantifying the multi-enzyme complex [20] was 254.50 ± 4.53 filter p aper u nit per millilitre (FPU/mL), 89.53 ± 0.43 U/mL and 12084.88 ± 169.33 U/mL for cellulase [21], cellobiase [21], and xylanase [22], respectively.

### 2.3. Enzymatic Pretreatment Process

The sulphite, eucalyptus, and thermomechanical pulp were cut into small pieces and hydrolysed using different enzyme loads: 5, 10, 20, and 40 FPU/grams of dry substrate (FPU/gdp). Consequently, 1 g of pulp was mixed in a solid–liquid ratio of 1:30 (*w*/*v*) with sodium citrate buffer 50 mM, pH 4.8 at 150 rpm for 1 h and 16 h [23]. At the end of the enzymatic pretreatment, the enzyme was denatured in a water bath at 100 °C for 5 min [20]. The sugars in the aliquots were determined by high-performance liquid chromatography (HPLC).

### 2.4. Characterisation of the Enzyme-Pretreated Fibres

#### 2.4.1. Chemical Composition

Non-pretreated and enzymatically pretreated pulps, dried to constant weight, were analysed for polysaccharide and total lignin content according to National Renewable Energy Laboratory protocols (NREL/TP-510-42618) [24]. Glucose and xylose concentrations were first quantified by HPLC (Agilent 1200, Palo Alto, CA, USA) equipped with a refractive index detector and an Aminex HPX-87H ion-exclusion column (Bio-Rad, 300 × 7.8 mm, 9 μm particles), using standards in the range of 0.1–4 mg/mL. These values were subsequently converted into glucan and xylan contents following the NREL protocol. Total lignin was determined by quantifying both acid-soluble lignin (ASL) and Klason lignin (KL) [24]. For ASL quantification, each sample was diluted with 4% (*w*/*w*) H_2_SO_4_ and measured at 205 nm using a UV–Vis spectrophotometer Libra S60 from Biochrom (Cambridge, UK). The solid residue obtained after hydrolysis was oven-dried at 105 °C and considered Klason lignin (KL). Each sample was analysed in triplicate using representative subsamples. Data are presented as the mean ± standard deviation, and statistical significance was assessed by a one-way ANOVA followed by Tukey’s post hoc test using Minitab Statistical Software 22.4.0.

#### 2.4.2. Morphological Characterisation

Fibre morphology changes resulting from enzymatic hydrolysis pulp were analysed using a MorFi Compact Techpap (Grenoble, France) equipped with a CCD video camera and MorFi v9.2 software. For each sample, four tests analysing 120,000 fibres were conducted. The fibre concentration was set at 25 mg/L, and parameters such as fibre length and diameter were measured [25].

The morphology of papers from non-pretreated and enzymatically pretreated pulps was analysed using a TESCAN Clara II ultra-high-resolution field-emission scanning electron microscope (FESEM, Madrid, Spain). Images were acquired in STEM mode using the transmitted electron detector. Samples were prepared on 200-mesh copper grids and stained with 1% uranyl acetate. At least three representative micrographs were obtained per sample.

### 2.5. Paper Preparation

The paper grammage was determined as the ratio of weight to area, and adjusted to 75 g/m^2^. Approximately 0.330 g of sulphite, eucalyptus, or thermomechanical pulp, either non-pretreated or enzymatically pretreated, was diluted in distilled water. The suspension was then dispersed using an Ultra-Turrax homogenizer Unidrive X 1000 (Baden-Württemberg, Germany) for 10 s at 10,000 rpm. The dispersed solution was subsequently vacuum-filtered using a 47 mm filtration assembly with glass support and a fine aluminium mesh (mesh size: 0.105 mm, wire diameter: 0.050 mm, fabric AISI-304 No. 180A, Ref. 23100180A). Finally, the produced wet cake was pressed and dried using a Rapid– Köthen automatic sheet forming machine (Österreich, Austria) at 90 °C during 1 min according to ISO 5269-2:2004 (E) (Pulps—Preparation of laboratory sheets for physical testing—Part 2: Rapid-Köthen method; International Organization for Standardization, Geneva, Switzerland, 2004). All the samples were conditioned at 23 °C and a relative humidity of 50% for 24 h before testing (ASTM D618 method).

### 2.6. Paper Characterisation

#### 2.6.1. Evaluation of Surface Physical Properties

Surface physical characteristics were evaluated on papers produced from non-pretreated and enzymatically pretreated pulps, prepared as described in Section 2.5. The thickness of the papers was measured using a digital micrometer LAF (Starrett, Athol, MA, USA). Air permeability (µm/Pa·s) was determined according to ISO 5636-5:2013, Paper and board—Determination of air permeance (medium range)—Part 5: Gurley method (International Organization for Standardization, Geneva, Switzerland, 2013).

Internal bond strength (J/m^2^) was assessed with a Scott-type tester following the TAPPI/ANSI T 569 om-22 method. Surface hydrophobicity was determined by measuring the static water contact angle (°) using a DSSA25 droplet shape analyser (Hamburg, Germany) equipped with Krüss Advance software. Measurements were performed at room temperature (~20 °C) with a data acquisition frequency of two measurements per second. All measurements were carried out in duplicate, and the results are reported as the mean values ± standard deviation. Statistical significance was assessed by a one-way ANOVA followed by Tukey’s post hoc test, using Minitab Statistical Software 22.4.0.

#### 2.6.2. Mechanical Analysis

Mechanical properties were evaluated on papers produced from non-pretreated and enzymatically pretreated pulps, prepared as described in Section 2.5. Tests were performed on an INSTRON 3340 universal testing machine equipped with a 100 N load cell. Paper strips (15 mm × 50 mm), cut in the machine direction, were conditioned at 23 °C and 50% relative humidity for 24 h prior to testing. Tensile strength (MPa) was measured at a crosshead speed of 100 mm/min. All measurements were carried out in duplicate, and the results are reported as mean values ± standard deviation. Statistical significance was evaluated by a one-way ANOVA, and Tukey’s post hoc test was applied using Minitab Statistical Software 22.4.0.

#### 2.6.3. FTIR Spectroscopy

The chemical changes after the enzymatic pretreatment were also studied using ATR-FTIR spectra (Bruker Alpha, Barcelona, Spain) equipped with a diamond crystal plate ATR MIR single-refection accessory. FTIR spectra were recorded in the range of 4000–400 cm^−1^, with a resolution of 8 cm^−1^ and 32 scans per sample. Paper samples were conditioned at 23 °C and a relative humidity of 50% for 24 h prior to analysis and placed directly on the ATR crystal. For each condition, two independent spectra were collected, and the average values were used to calculate the indices.

#### 2.6.4. Crystallinity Index

The total crystallinity index (*TCI*; Equation (1)) was determined from the ATR-FTIR spectra obtained as described in Section 2.6.3, for papers produced from non-pretreated and enzymatically pretreated pulps, following the method reported by Narhoglu et al. [26]. In this analysis, the band at 1378 cm^−1^ corresponds to C–H deformation in cellulose, while the peak at 2900 cm^−1^ is associated with C–H stretching in methyl (–CH_3_) and methylene (>CH_2_) groups of cellulose [27,28,29].

(1) $$TCI=\frac{A\;1378\;{cm\;}^{-1}}{A\;2900\;{cm\;}^{-1}}$$

Similarly, changes in cellulose crystallinity were evaluated using the lateral order index (*LOI*), calculated from the absorbance at 1437 cm^−1^, corresponding to symmetric CH2 bending in the crystalline regions, and that at 897 cm^−1^, corresponding to glycosidic bond β-(1,4) in cellulose and associated with the amorphous regions of cellulose (Equation (2)) [20,30].

(2) $$LOI=\frac{A\;1437\;{cm}^{-1}}{A\;897\;{cm}^{-1}}$$

## 3. Results and Discussion

### 3.1. Characterisation of Enzyme-Pretreated Pulps

The initial polysaccharide and lignin composition of the different industrial pulps used in this study is presented in Table 1. In this respect, it is observed that the sulphite pulp was composed mainly of cellulose. Sulphite pulp followed a sodium-based acidic sulphite pulping process, to fraction the lignin present in resinous wood such as spruce softwood [31]. During this phase, lignin undergoes sulfonation (water-soluble), and some hemicellulose is extracted. However, highly acidic firing conditions in resinous wood can lead to condensation reactions of lignin over delignification which could explain the 1.45 ± 0.29 of total lignin present on sulphite pulp [32]. On the other hand, the eucalyptus pulp presented a composition rich in glucan (80%) and xylan (20.5%), and low composition in total lignin (~1%) while the thermomechanical pulp contained a holocellulose composition (glucan and xylan) close to ~66.56 and ~20% of total lignin. This difference could be associated with the type of pretreatment as well as the raw source used. The process of obtaining thermo-mechanical pulp involves three processing stages (wood chip pretreatment, refining and pulp processing), in which wood chips are washed with steam in two stages at atmospheric pressure, first at 100 °C and later at 70–85 °C. Finally, a pulp process is carried out to improve the strength and optical properties [33].

Therefore, the difference in the industrial pulp composition of this study is mainly related to the source (raw pulp) and the type of pretreatment applied during its processing. In this aspect, the variance in their composition can generate a modification in their mechanical, chemical, and morphological properties.

**Figure 1:**
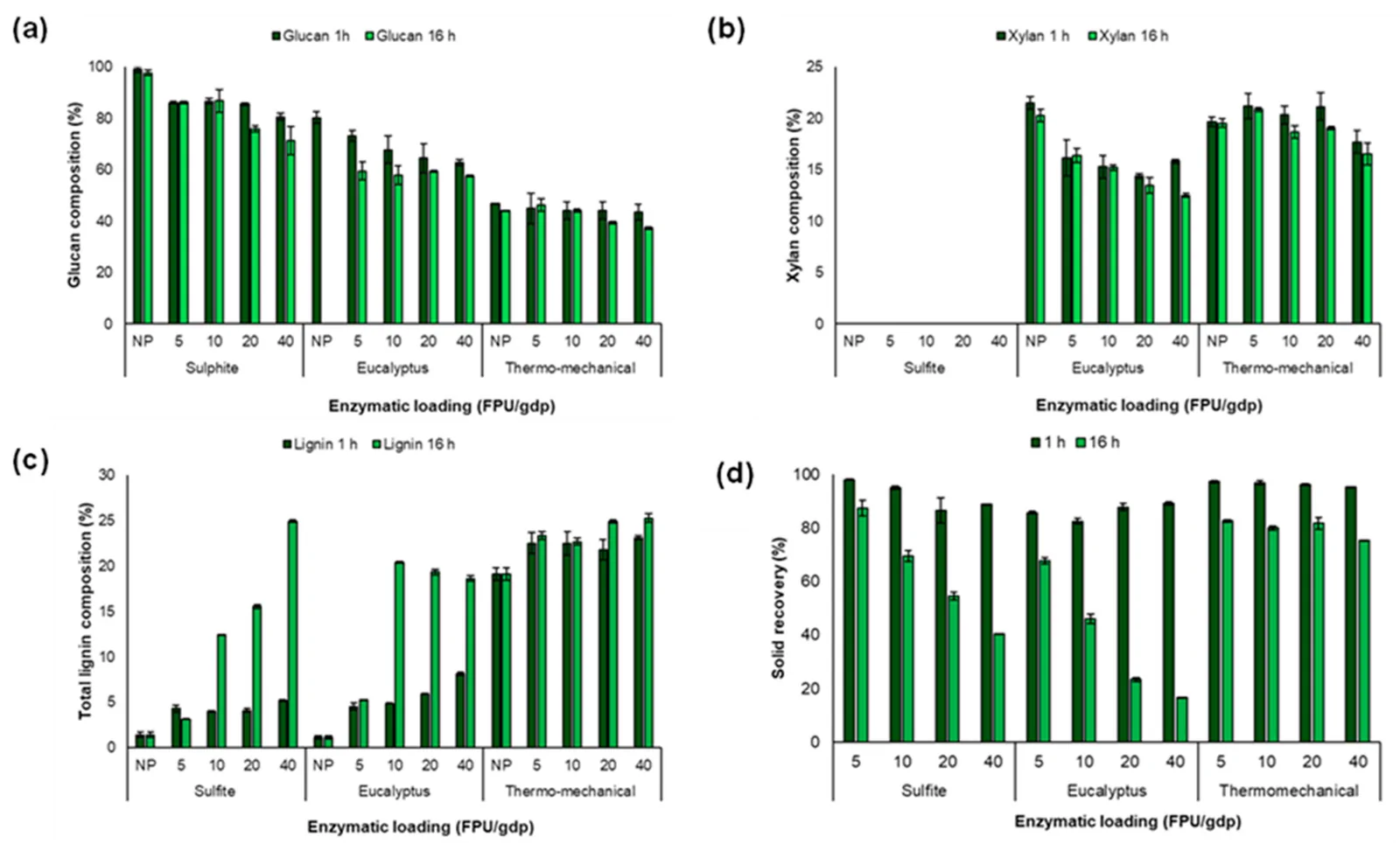
Lignocellulosic composition of sulphite, eucalyptus, and thermomechanical pulps after the enzymatic hydrolysis using different enzymatic loadings per gram of dry pulp (FPU/gdp), (**a**) glucan composition, (**b**) xylan composition, (**c**) total lignin composition and (**d**) solid recovery percentage, after 1 h (dark green bars) and (**b**) 16 h (green bars) pretreatment. NP: n on-pretreated pulps; ST: solid recovery.

The initial composition of the different industrial pulps and its composition after enzymatic hydrolysis at 1 h and 16 h of time reaction are shown in Figure 1. The results demonstrated no significant changes (see Table S1) in sulphite pulp composition until the more severe operational conditions were applied, 16 h and an enzymatic load of 20 or 40 FPU/g, reducing the glucan content to 76.01% and 71.39%, respectively (Figure 1a). This is explained by the fact that the sulphite pulp is composed mainly of glucose chains aligned into the very compact fibres called microfibrils, which are tightly packed against each other generating a dense and ordered (crystalline) structure [34].

Additionally, the composition of eucalyptus pulp was affected according to the severity of the pretreatment, including reaction time and enzyme loading. Thus, it was observed that the hardiest treatment, 16 h and an enzyme loading of 40 FPU/gdp, reduced the glucan content to 60%. In this regard, it can be suggested that the degradation of glucan and xylan (Figure 1b) to their respective monomers can correlate to the enzyme cocktail used in this study. Since, Cellic CTec2 enzyme contains xylanases as the accessory enzyme [20], such as endoxylanase enzymes, which catalyse the random hydrolysis of the internal β-1,4 glycosidic linkages between the xylose residues in the xylan backbone, releasing xylooligosaccharides. Therefore, the action of endoxylanase enhanced the accessibility of enzymes such as endoglucanases to the amorphous regions of cellulose, which promoted cellulose hydrolysis [35].

However, only small differences (Table S2) were observed in the hydrolysis of xylan in eucalyptus pulp under the different enzyme loadings (5–40 FPU/gdp). This may be attributed to the presence of more resistant xylan on the pulp surface, i.e., linear xylan resulting from the removal of acetyl and glucuronic acid side groups [36]. In addition, it has been reported that the xylan dissolved during the Kraft process can precipitate or be re-adsorbed onto the fibre surface. Such rearrangement of xylose units may promote tighter packing of xylan chains and their co-crystallisation with cellulose, leading to decreased solubility, increased crystallinity, and the formation of aggregates in the biomass [19]. These structural changes could limit enzyme accessibility to the substrate, thereby resulting in non-significant effects of reaction time or enzyme loading.

Otherwise, higher enzyme loadings (≥20 FPU/gdp) and longer reaction times (16 h) were required for the thermomechanical pulp to generate a significant effect on glucan composition (Figure 1a). This may be related to the lignin content in the initial thermomechanical pulp (Figure 1c and Table S3). Lignin is a biopolymer that can adsorb enzymes during enzymatic hydrolysis, leading to the formation of lignin–enzyme complexes and reducing enzyme accessibility to polysaccharides [23,37]. Moreover, softwood lignin (high proportion of guaiacyl units) has been found to inhibit to a greater extent the enzymatic hydrolysis compared to hardwood lignin (richer in both guaiacyl and syringyl monolignols) [37]. Therefore, it is likely that the low efficiency of enzymatic blend in thermomechanical pulp from pine (softwood) was due to the high presence of guaiacyl units in lignin.

Lignin interacts with cellulases through hydrophobic interactions, electrostatic forces, and van der Waals forces [38]. Particularly, hydrophobic interactions are the main force driving the association between lignin and cellulases, and entropy, being highly dependent on temperature, may play a dominant role in facilitating the adsorption process through these interactions [39]. However, some alternatives are purposed to mitigate lignin– enzyme adsorption in enzymatic hydrolysis such as the use of lignin-block additives (non-ionic surfactants, biosurfactants, lignin-modified polymers, and non-catalytic proteins), cellulases protectors (lignosulfonate and/or soluble lignin) [38] or engineer enzymes that are less prone to binding with insoluble lignin [37]. Other options also include the addition of additives in the pretreatments namely surfactants (Tween 80, dodecylbenzene sulfonic acid and PEG 4000), that increase delignification and reduce non-productive adsorption between residual lignin and cellulase enzymes, or carbocation scavengers (nucleophilic compounds like naphthol derivatives, phenolic acids or mannitol), which act as nucleophiles, focus on the C_α_ and minimize the occurrence of lignin repolymerization [38].

In addition, Figure 1d shows material recovery, which was mainly influenced by reaction time (Table S4). After 1 h of enzymatic pretreatment, overall solid recoveries of 85% for sulphite and eucalyptus pulps, and 95% for thermomechanical pulp were obtained using the highest enzyme loading (40 FPU/gdp). In contrast, after 16 h of pretreatment, solid recoveries decreased to 40%, 17%, and 75% for sulphite, eucalyptus, and thermomechanical pulps, respectively.

These results highlight that lignin content can either facilitate or hinder the efficiency of polysaccharide hydrolysis. Therefore, the selection of specific pretreatment conditions should be guided by the intended objective.

The results of the morphology characterisation using MorFi equipment for the control pulps (non-pretreated) and the enzymatically pretreated sulphite, eucalyptus, and thermomechanical pulps are presented in Table 2.

The fibre length distribution of enzymatic pretreated pulps was modified according to the reaction time and enzymatic loading. Sulphite, eucalyptus, and thermomechanical pulps exhibited a moderate reduction in of the mean fibre length (307 µm, 330 µm, and 322 µm, respectively), corresponding to decreases of 36%, 33.4%, and 2.13% under the highest enzymatic load (40 FPU/gdp) after 1 h of reaction. After 16 h of pretreatment at 40 FPU/gdp, the mean fibre length decreased to 157 µm, 142 µm, and 240 µm, representing reductions of ~70% for sulphite and eucalyptus pulps and ~30% for thermomechanical pulp compared with the non-pretreated samples. These findings suggest that the characteristic composition of sulphite and eucalyptus pulps enhances accessibility, facilitating enzyme action and improving polysaccharide hydrolysis.

No significant differences in mean fibre diameter were observed after enzymatic pretreatment, suggesting that reaction time and enzymatic loading do not affect this parameter. These results are consistent with Chen et al. [10], who reported that enzymatically pretreated cellulose fibres showed no change in width after 5–11 h of reaction using cellulases as catalysts

In addition, these results are agreed with those reported by Mazega et al. [25], which indicate a slight change in eucalyptus fibre morphology after enzymatic pretreatment using endoglucanases. However, the authors confirmed that after enzymatic pretreatment the fibres with a cross-over backbone and fibre kinking were modified to a similar blooming tree structure.

Based on these results, it is evident that pretreatment conditions using commercial enzymes should be optimized to meet the specific requirements of the target products without compromising their final quality [17].

### 3.2. Structure and Properties of Papers from Enzyme-Pretreated Pulp

**Figure 2:**
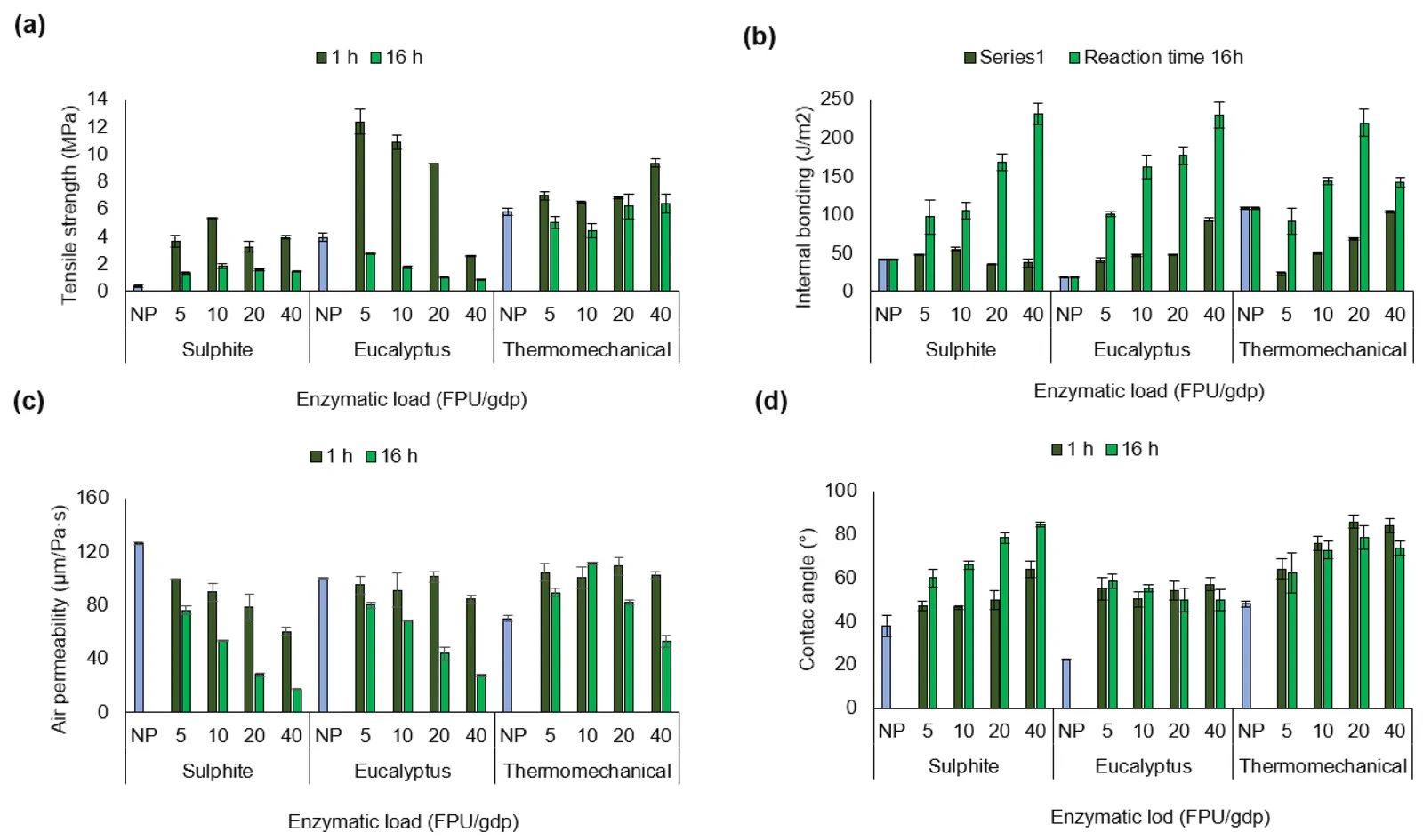
(**a**) Tensile strength (MPa), (**b**) internal bonding (J/m^2^), (**c**) air permeability (µm/Pa·s), and (**d**) contact angle (°) of papers from non-pretreated pulp (NP; blue bars) and enzymatically pretreated pulps, after 1 h (dark green bars) and 16 h (green bars) of reaction time under different enzymatic loadings.

Tajik et al. [40] reported that paper strength is related to the number of bonds between fibres during fibre network consolidation and drying, as well as the intrinsic fibre strength. In this regard, Figure 2 illustrates the tensile strength, internal bonding, air permeability, and contact angle results of papers from non-pretreated and enzymatic pretreated pulps.

The enzymatic pretreatment significantly improved (Table S5) the tensile strength of paper made from sulphite pulp (Figure 2a). Here, the increase in tensile strength under the pretreatment condition of 1 h and 10 FPU/gdp represented an approximately 14-fold improvement over the initial value. However, extending the reaction time to 16 h and increasing the enzyme load to 40 FPU/gdp did not produce a beneficial effect. Lecourt et al. [3] proposed that endoglucanase activity weakens the fibre structure by creating weak points or kinks, leading to fibre cutting and fibrillation during mechanical refining. This is consistent with the reduction in fibre length observed in Table 2 (from 476 µm to 157 µm). Fibre length, intrinsic fibre strength, specific bond strength, the strength of bonded areas, sheet formation, and the distribution of applied stress are all considered influential factors determining paper strength [41].

The tensile strength of papers prepared from enzymatically pretreated eucalyptus pulp showed a clear positive effect compared to non-pretreated pulp. The most favourable improvement was observed under the operational condition of 1 h and 5 FPU/gdp, representing approximately a threefold increase over the initial value. However, extending the reaction time to 16 h led to a decrease in tensile strength, with higher enzyme loadings resulting in progressively lower values. Notably, eucalyptus pulp exhibited the highest tensile strength compared to sulphite and thermomechanical pulp. This may be related to its cellulose and xylan composition, as well as to the type of enzyme complex applied. According to Ribeiro-Dias et al. [17], enzyme combinations with xylanase activity equal to or exceeding endoglucanase activity can produce nanocellulose crystals with enhanced properties in bleached eucalyptus Kraft pulp, including crystallinity, thermostability, uniformity, suspension stability, and aspect ratio. The beneficial effects of these accessory enzymes are attributed to their hydrolytic action on xylan and cellulose, rather than to swelling of the cellulose fibres or changes in fibre porosity.

In contrast, the tensile strength of papers made from thermomechanical pulp increased under more severe operational conditions, specifically higher enzymatic loadings. This improvement may be attributed to changes in chemical composition following pretreatment, as well as to the reported increase in lignin content (Figure 1c). In this sense, lignin is a polymer composed of spherical nanoparticles with intense interactions inside that after pretreatment can repolymerise and strengthen the interactions between polysaccharides through β-O-4 aryl ester bonds and 4-O-methylglucuronic acid bonds randomly distributed along the xylan chains [23]. Therefore, residual lignin on the surface of the treated cellulosic fibres may be activated and act as a natural adhesive [9]. In this context, the interlocking of lignin within the macro- and micro-irregularities of the fibre surface may contribute significantly to fibre adhesion, thereby enhancing the mechanical properties of the paper.

Based on the results, sulphite pulp exhibited lower tensile strength compared to eucalyptus and thermomechanical pulps. According to Klemm et al. [42], this may be attributed to the fact that sulphite fibres are more prone to fibrillation and delamination than kraft pulps or pulps with a high hemicellulose content. The results obtained are consistent with those reported by Bastida et al. [43] who observed a maximum tensile strength of 12.5 MPa in membranes fabricated from cellulose acetate reinforced with cellulose nanofibres. In addition, the tensile strength obtained (11.20 MPa) exceeds the values reported by Wang et al. [44] for papers prepared from textile waste as self-reinforcing cellulose fibres, without the addition of CNFs.

This study introduces a greener and more straightforward strategy for producing cellulose as a reinforcing material. Previous research has demonstrated that enzymatic pretreatments enhance fibre fibrillation and lower the specific energy demand during refining [4,45]. For instance, Mohlin and Pettersson [45] reported the energy savings of 40–70% (45–65 kWh/t) when enzymatic pretreatment was applied prior to industrial disc refining, thereby reducing the need for energy intensive mechanical treatments. Moreover, enzymatic processes can foster cleaner and more sustainable operations by limiting the use of harsh chemicals commonly employed in conventional pretreatments [46]. Similarly, Tanveer et al. [47] highlighted that in papermaking, enzymes can substitute chemically intensive steps, leading to reductions in energy consumption and operational costs while improving fibre quality. Collectively, these findings underscore the potential of enzymatic catalysis as a promising approach to decrease energy requirements and mitigate the environmental footprint of pulp and paper production.

The internal bond strength of papers prepared from enzymatically pretreated and non-pretreated pulps is shown in Figure 2b. Enzymatic pretreatment increased the internal force of the fibres at both 1 h and 16 h of reaction, indicating that all enzyme loadings were effective. However, the use of the higher operational condition (16 h and 40 FPU/gdp) generated a significant improvement compared to the other conditions (see Supplementary Table S6). For example, the internal bond strength of sulphite pulp fibres increased from an initial 42 J/m^2^ to 231 J/m^2^, representing a 5.5-fold improvement. The internal bond strength of eucalyptus pulp increased from 18 J/m^2^ to 230 J/m^2^, representing an improvement of nearly 13-fold compared to non-pretreated pulp. Similarly, fibres from thermomechanical pulp increased from 108 to 220 J/m^2^ following pretreatment for 16 h at 20 FPU/gdp. A possible explanation is related to the enzymatic pretreatment, as the enzymes break specific bonds within the fibres (β-1,4-glycosidic bonds, β-O-4 aryl ester bonds, and xylan chains of 4-O-methylglucuronic acid), potentially facilitating molecular-level contact between fibres through adhesion via van der Waals forces, hydrogen bonds, and electrostatic interactions [9]. Similarly, the filtration process and high-vacuum drying of the paper during processing may promote fibre consolidation, thereby increasing internal bond strength [48,49].

Figure 2c shows that the air permeability of non-pretreated pulps differs according to the type of industrial pulp as follows: sulphite > eucalyptus > thermomechanical (Table S7), which may correlate to differences in chemical composition. Sulphite pulp consists mainly of glucose chains aligned into compact fibres packed closely together, forming a dense and orderly structure. This arrangement results in small inter-fibre spaces, limiting the passage of air and other gases. In contrast, eucalyptus and thermomechanical pulps contain more amorphous components, such as xylan and lignin, which can lead to a more porous material [34]. According to the results obtained the operational condition plays a crucial factor, since the use of longer pretreatment (16 h) or a higher enzyme loading allows for a more severe clogging between fibres, while a shorter treatment (1 h) can maintain a paper with a medium porosity. Moreover, the enzyme complex can alter cellulose fibre swelling and porosity by breaking specific bonds within the lignocellulose [20].

This study also assessed the surface hydrophilicity of the papers by measuring the water contact angle (Figure 2d). The results indicated an increase in the hydrophobicity of the surface of papers made from enzymatic pretreated pulps, with respect to the non-pretreated pulps. For instance, paper made from sulphite pulp pretreated for 16 h at 40 FPU/gdp reached a contact angle of 85° ± 1.0°, whereas paper from non-pretreated sulphite pulp exhibited a contact angle of 38° ± 4.8°. No significant differences (see Table S8) in the hydrophobicity of papers made from eucalyptus pulp were observed after 1 h of pretreatment, regardless of the enzymatic loading. Nevertheless, increasing the enzymatic load (from 5 to 40 FPU/gdp) and extending the reaction time to 16 h resulted in a decrease in the contact angle of papers made from eucalyptus and thermomechanical pulps. This effect may be attributed to increased fibrillation and changes in porosity within the pulp matrix following enzymatic pretreatment.

Therefore, alterations in the interconnected fibrous structure of papers made from pretreated pulp can either hinder or promote water diffusion through the fibre matrix. This effect is driven by increased fibrillation, fibre flexibility, or fine generation, which ultimately leads to the formation of a denser paper [43].

In this context, the air permeability results (reflecting matrix porosity) can be correlated with the contact angle measurements. Capillarity, which describes the ability of a liquid to flow through narrow spaces without external forces, arises from the surface tension of the liquid and its adhesion to the walls of a porous or capillary material [50].

### 3.3. Morphological and Chemical Characterisation of Enzyme-Pretreated Pulp Papers

**Figure 3:**
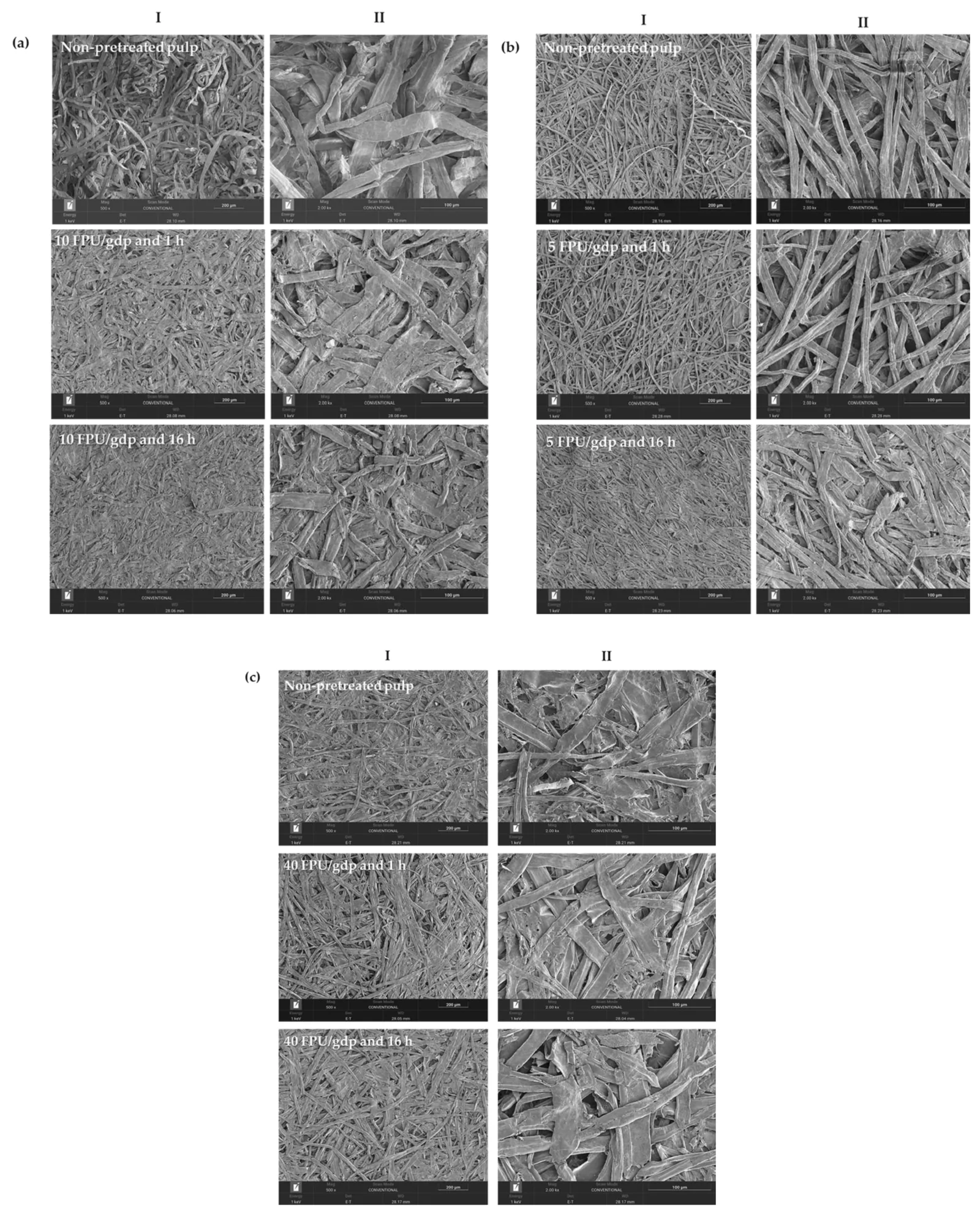
Surface SEM micrographs of papers produced using (**a**) sulphite, (**b**) eucalyptus, and (**c**) thermo-mechanical pulps under different operational conditions. Images (**I**,**II**) correspond to different magnifications. Scale bars: I = 200 µm; II = 100 µm.

Scanning electron microscopy (SEM) micrographs (Figure 3) were obtained from papers produced using sulphite, eucalyptus, and thermomechanical pulps, both with and without enzymatic pretreatment, under varying enzyme loadings and reaction times.

Papers produced from non-pretreated sulphite, eucalyptus, and thermomechanical pulps exhibited a fibrous and porous structure with frequent voids and empty spaces, which is consistent with the results obtained for air permeability. In contrast, the micrographs of papers prepared from enzymatically pretreated pulps revealed partial filling of the fibre network voids and greater retention of fibre fines (Figure 3a–c, section I). Moreover, after 16 h of pretreatment, a more pronounced fibre reorganisation was observed (Figure 3a–c, section II). As the enzymatic treatment duration increased from 1 to 16 h, microfibril porosity decreased, resulting in a more uniform, smooth, and dense paper surface.

The fibre aggregation observed in the micrographs could also be associated with changes in fibre length and thickness (Table 2; fibre morphology, MorFi Compact Techpap), as well as with the air permeability results (Figure 2), which decreased upon enzymatic pretreatment. Furthermore, structural and physical property measurements showed increases in tensile strength and internal bonding (Figure 2a,b), indicating denser sheet formation and improved fibre–fibre contact. This effect may be attributed to enzymatic pretreatment, since cellulose microfibrils contain abundant hydroxyl groups that enhance reactivity and flexibility, thereby promoting fibre consolidation and strengthening inter-fibre bonds [51].

Additionally, the SEM micrographs of papers from enzymatically pretreated pulps exhibited more frequent fibre agglomeration compared with papers from non-pretreated pulps. This phenomenon may be related to chemical composition, as xylan has been reported to promote the coalescence of cellulose nanofibres (CNFs) [51]. This suggests that hemicelluloses on the CNF surface could provide steric hindrance or electrostatic repulsion between nanofibrils, thereby enhancing dispersion stability [19,52].

Fourier transform infrared spectroscopy (FTIR) was used to analyse the structural chemical differences between the treated and non-treated fibres. The expected structural changes in characteristic FTIR peaks of papers prepared from non-pretreated pulps (control) and under the best enzymatic hydrolysis conditions studied are included in Table 3.

**Figure 4:**
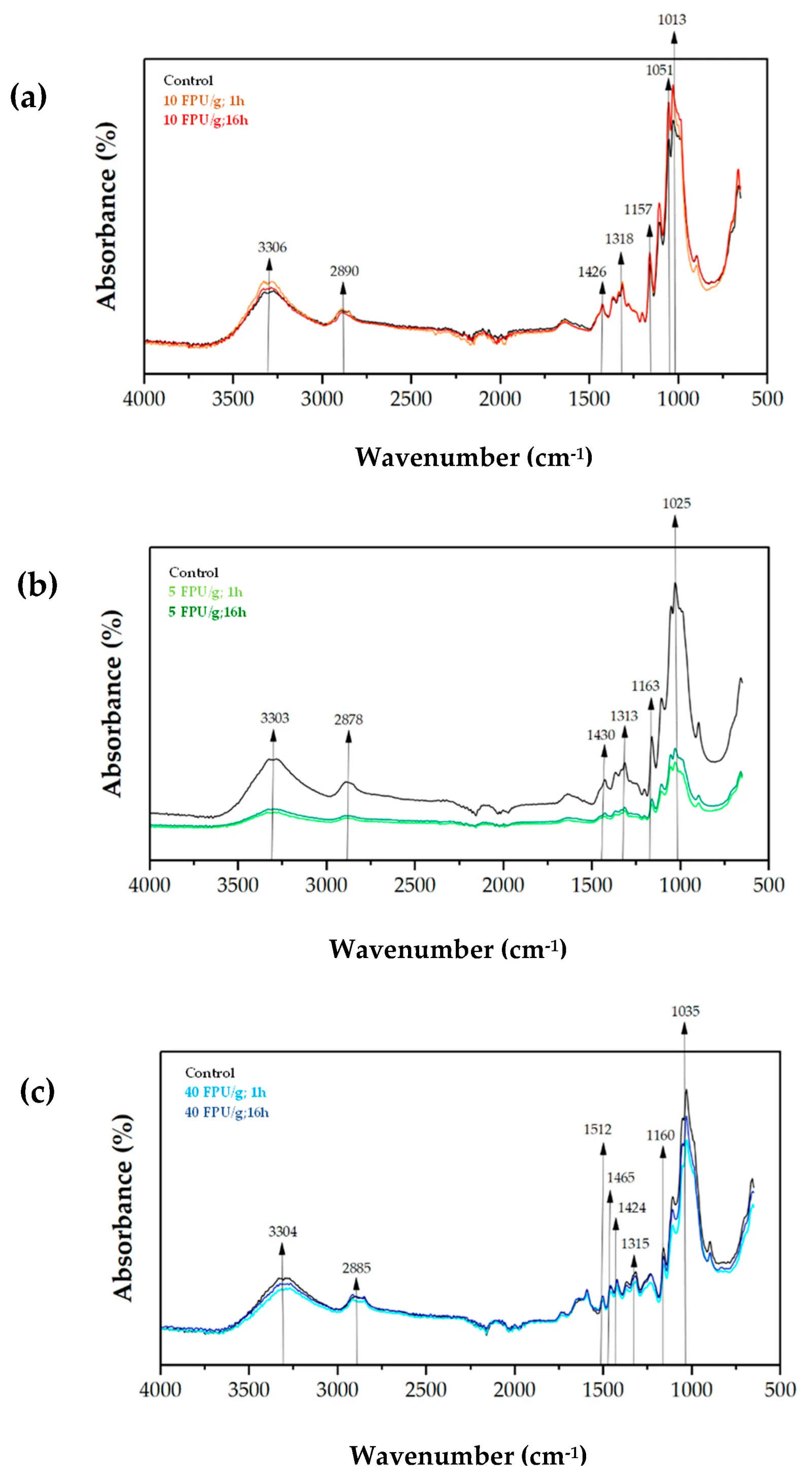
ATR-FTIR spectra of papers from (**a**) sulphite pulp, (**b**) eucalyptus pulp, and (**c**) thermomechanical pulp. Black line represents control samples, which means non-pretreated pulps, while coloured lines indicate the enzymatically pretreated fibres.

The ATR-FTIR spectra of papers from the different pulps, without pretreatment (control) and after enzymatic pretreatment at different operating conditions are included in Figure 4.

The papers produced from non-pretreated sulphite pulp (control) and enzymatic pretreatment exhibited similar FTIR spectra (Figure 4a). The increase in the absorbance of the peaks 3306 cm^−1^, 2890 cm^−1^, 1426 cm^−1^, 1318 cm^−1^ and 1157 cm^−1^ was noticed. This may represent an increase in the O–H groups due to the defibrillation of the cellulose fibres by the enzymes [28,54]. On the other hand, the peaks at 1051 cm^−1^ and 1013 cm^−1^ may correlate with the increase in lignin after hydrolysis of cellulose into non-recoverable monomers [28,55].

In contrast, the reduction in the absorbance pattern of the paper composed by enzymatic pretreated eucalyptus pulps with respect to the non-pretreated is apparent in Figure 4b. The results on chemical composition may support this observation, since glucan and xylan content decreased after enzymatic pretreatment. From Table 3, the peak at 1313 cm^−1^ correlates to the reduction in xylan content, while the reduction in cellulose (glucan) can be associated to the peaks at 3303 cm^−1^, 2878 cm^−1^, 1430 cm^−1^, 1163 cm^−1^ and 1015 cm^−1^ [28,53]. Additionally, the decrease in absorbance at peaks 3303 cm^−1^, and 2878 cm^−1^ suggests a change in the structure of the cellulose fibres, i.e., a reduction in the availability of O–H groups which indicates a less recalcitrant fibre [44].

The FTIR analysis of thermomechanical pulp papers produced from enzymatically pretreated pulp (Figure 4c) did not show significant differences compared with the control paper (non-pretreated pulp). This observation is consistent with the results discussed in Figure 1. However, the slight reductions in the peaks at 1424 cm^−1^, 1315 cm^−1^, and 1160 cm^−1^ may be related to a decrease in xylan (hemicellulose) content. In contrast, the peaks at 3304 cm^−1^, 2885 cm^−1^, and 1035 cm^−1^, associated with cellulose (glucan), suggest changes in the recalcitrance and deconstruction of the cellulose fibres [20]. Furthermore, the peaks at 1512 cm^−1^ and 1465 cm^−1^, corresponding to C=C vibrations of guaiacyl aromatic skeletons of lignin and C–H deformations (methyl and methylene), respectively [20], indicate the presence of lignin after enzymatic hydrolysis, which is consistent with the compositional analysis described above.

The ratio of the CH_2_ asymmetric stretching vibration peaks seen at about 2900 cm^−1^ and the C–H asymmetric deformation vibration at about 1378 cm^−1^ gives an insight into the total crystalline index (TCI) (A1378/A2900). The ratio at 1437 cm^−1^ in-plane scissoring (symmetric bending) and CH deformation in β-glycosidic linkages at 897 cm^−1^ reveals the lateral order index (LOI) (A1437/A897). The TCI is proportional to the crystallinity degree of cellulose, while the LOI is correlated to the overall degree of order in cellulose [26,53,56]. The obtained values are given in Table 4.

On one hand, an increase in the TCI value was observed after modification of the chemical and physical structure of sulphite and eucalyptus pulp by enzymatic hydrolysis. Under more severe operating conditions such as prolonged reaction times, cellulose crystallinity was slightly enhanced for the sulphite pulp rising to 1.24 and remaining at 1.21 for the eucalyptus pulp. In contrast, a decrease in the TCI value was observed for thermomechanical pulp following enzymatic hydrolysis. This reduction may be explained by the chemical composition of the thermomechanical pulp, which likely promotes a synergistic action between cellulase and xylanase enzymes, leading to more extensive cellulose degradation and, consequently, a greater decomposition of the crystalline phase of the original fibre.

The lateral order index (LOI) of papers produced from both non-pretreated and enzymatically pretreated pulps was determined to assess qualitative changes in the crystallinity of the cellulose structure [20,30]. The LOI values of sulphite pulp increased to 0.50 from 0.45 in the sample pretreated with 10 FPU/gdp for 1 h, and to 0.47 from 0.43 in the eucalyptus sample pretreated with 5 FPU/gdp for 1 h. However, the application of more severe conditions, such as prolonged reaction times, reduced the crystalline fraction. In contrast, the LOI value of thermomechanical pulp increased after enzymatic pretreatment, and also when longer hydrolysis times were applied. This effect may be explained by the fact that prolonged enzymatic treatment can promote a more extensive hydrolysis of amorphous components, such as hemicellulose (xylan), which in turn may increase the contact area with amorphous cellulose (glucans) through the existing β-(1→6) branches. These branches enable hemicellulose to “anchor” to cellulose and other matrix components, thereby facilitating a more efficient interaction between enzymes and the substrate and ultimately enhancing the crystalline fraction of the solid material (pure cellulose) [57].

In summary, this work demonstrated promising the results of enzymatic hydrolysis, which notably enhanced the mechanical and surface properties of the pulps with potential application as a reinforced agent in packaging. Likewise, the enzymatically treated pulps could also be tested in composites and hydrogels formulations [58,59].

Despite this fact previously mentioned, there are still challenges for this technology to be implemented on a large scale such as the high cost of enzymes, slow biochemical reaction of cellulases or limited recovery of enzymes [60]. Additionally, other technical and economic factors should be considered the cost of enzyme production (specific bioreactors, raw materials and purification steps), high enzyme loads, and the specificity of enzyme used [61]. Therefore, further research is still required to optimise the enzymatic hydrolysis process for large-scale implementation.

## 4. Conclusions

The effect of a cellulase- and xylanase-rich enzyme cocktail was evaluated on three industrial pulps: sulphite, bleached kraft eucalyptus, and thermomechanical pine. Enzymatic hydrolysis degraded glucan and xylan into monomers, with eucalyptus and pine pulps showing progressive changes, while sulphite pulp remained stable under mild conditions. In thermomechanical pulp, high lignin content hindered enzyme access due to lignin–enzyme complex formation.

The enzymatic pretreatment induced surface fibrillation, fibre shortening, and fine formation in all industrial pulps. Morphological characterization revealed fibre aggregation and structural modifications in the papers produced from enzymatically pretreated pulps.

According to the results, eucalyptus pulp enzymatically pretreated at 5 FPU/gdp for 1 h exhibited the highest tensile strength (12.40 ± 0.90 MPa). However, when considering overall performance, sulphite pulp showed the most consistent improvement across mechanical properties. In particular, internal bonding (230.90 ± 13.50 J/m^2^), air permeability (17.10 ± 0.50 µm/Pa·s), and contact angle (85.0° ± 1.0) were enhanced under longer reaction times (16 h) and higher enzymatic loadings (40 FPU/gdp), likely due to the formation of shorter fibres that agglomerated more effectively through chemical interactions. Moreover, tensile strength in sulphite pulp also improved (5.37 ± 0.04 MPa) at 10 FPU/gdp and 1 h. Therefore, while eucalyptus pulp at 5 FPU/gdp and 1 h maximized tensile strength, sulphite pulp enzymatic pretreatment can be considered the best overall performer, with the best conditions depending on the specific property targeted. Conversely, thermomechanical pulp did not show competitive results compared to the other pulps, which could be attributed to its high lignin content that hindered efficient enzymatic hydrolysis due to interlocking within the macro- and micro-irregularities of the fibre surface.

Therefore, enzymatic hydrolysis has shown clear potential in improving pulp properties; however, its industrial application still faces several challenges. These include the absence of cost–benefit analysis and limited comparisons with other enzyme systems under identical conditions. Additionally, technical and economic barriers such as high enzyme costs, slow reaction rates, and low recovery efficiency must be addressed. Future research should focus on overcoming these limitations, optimising process parameters (solid: liquid ratio, catalytic time, type of enzymes, the use of immobilized enzymes), and validating scalability to ensure practical viability.

## Figures and Tables

**Table 1:** Polysaccharide and lignin composition of different cellulose pulps (KL: Klason lignin; ASL: a cid-soluble lignin).

	Polysaccharide (%)		Lignin (%)		
Cellulose Pulp	Glucan	Xylan	KL	ASL	Total Lignin
Sulphite	99.06 ± 1.11 ^a^	0.00 ± 0.00 ^b^	1.28 ± 0.58 ^b^	0.17 ± 0.00 ^b^	1.45 ± 0.29 ^b^
Eucalyptus	80.03 ± 2.45 ^b^	20.49 ± 0.62 ^a^	0.88 ± 0.32 ^b^	0.26 ± 0.01 ^a^	1.14 ± 0.16 ^b^
Thermomechanical	46.86 ± 0.03 ^c^	19.70 ± 0.46 ^a^	18.99 ± 1.37 ^a^	0.12 ± 0.01 ^c^	19.11 ± 0.69 ^a^

Different letters within the same column indicate significant differences (ANOVA followed by Tukey’s test, *p* < 0.05).

**Table 2:** Fibre length and width distributions for the untreated and enzyme-pretreated pulps at different enzyme charge and treatment times.

Reaction Time (h)	Type of Pulp	Enzymatic Loading (FPU/gdp)	Fibre Length (µm)	Fibre Width (µm)
1	Sulphite	Non-pretreated	476.0 ± 10.0	23.0 ± 0.2
		5	468.0 ± 4.0	23.0 ± 0.3
		10	432.0 ± 16.0	23.0 ± 0.1
		20	416.0 ± 1.0	23.0 ± 0.0
		40	307.0 ± 14.0	23.0 ± 0.0
	Eucalyptus	Non-pretreated	495.0 ± 2.0	17.0 ± 0.1
		5	477.0 ± 1.0	17.0 ± 0.1
		10	474.0 ± 1.0	17.0 ± 0.1
		20	396.0 ± 2.0	17.0 ± 0.0
		40	330.0 ± 3.0	17.0 ± 0.1
	Thermo-mechanical	Non-pretreated	330.0 ± 14.0	24.0 ± 1.0
		5	320.0 ± 4.0	23.0 ± 0.1
		10	320.0 ± 1.0	23.0 ± 0.1
		20	324.0 ± 2.0	24.0 ± 0.1
		40	322.0 ± 11.0	24.0 ± 0.1
16	Sulphite	5	285.0 ± 4.0	21.0 ± 0.1
		10	202.0 ± 1.0	21.0 ± 0.01
		20	164.0 ± 0.01	21.0 ± 0.1
		40	157.0 ± 1.0	21.0 ± 0.1
	Eucalyptus	5	264.0 ± 4.0	18.0 ± 0.1
		10	203.0 ± 2.0	18.3 ± 0.1
		20	155.0 ± 2.0	18.0 ± 0.1
		40	142.0 ± 1.0	18.0 ± 0.3
	Thermo-mechanical	5	264.0 ± 0.01	22.0 ± 0.1
		10	281.0 ± 11.0	22.0 ± 0.1
		20	246.0 ± 1.0	22.3 ± 0.1
		40	240.0 ± 5.0	22.4 ± 0.3

**Table 3:** Functional groups by Fourier transform infrared spectroscopy (FTIR) spectral bands from paper produced by enzymatic pretreated pulp and non-pretreated pulp. SP: sulphite pulp; eucalyptus pulp; TMP: thermo-mechanical pulp.

Pulp Type	Wavenumber (cm^−1^)	Prediction of Functional Groups	Reference
SP	1013	Stretching: C–O, plane deformation of C–H in lignin	[20]
EP	1025		
SP	1051	Stretching vibration in C–OH and deformation of C–O in lignin	[20]
EP	1313	Wagging in CH_2_, stretching in C–O of substituted aromatic units in cellulose	[20]
SP	1318		
SP	1318	C–H_2_ wagging in cellulose	[53]
EP	1313		
EP	1430	Absorption of CH_2_ bending vibration in cellulose	[28]
SP	1426	Bending vibration in symmetric CH_2_, carboxyl group symmetric stretching band and deformation C–H in cellulose and hemicellulose	
TMP	1424		
TMP	1465	Deformation of C–H of methyl and methylene in lignin	[20]
SP	2890	Stretching vibration on symmetric and asymmetric C–H of methyl (–CH_3_) and methylene (>CH_2_) of cellulose	[20]
EP	2878		
TMP	2885		
SP	3306	Stretching and vibration of O–H of D-glucopyranose backbone in cellulose	[44]
EP	3303		
TMP	3304		

**Table 4:** Total crystalline index (TCI) and lateral order index (LOI) obtained from the FTIR analysis of the papers prepared from different pulps.

	Crystallinity	
Sample	TCI (A_1378 cm_^−1^/A_2900 cm_^−1^)	LOI (A_1437 cm_^−1^/A_897 cm_^−1^)
Sulphite non-pretreated	1.13	0.45
Sulphite 10 FPU/gdp; 1 h	1.14	0.50
Sulphite 10 FPU/gdp; 16 h	1.24	0.45
Eucalyptus non-pretreated	1.11	0.43
Eucalyptus 5 FPU/gdp; 1 h	1.22	0.47
Eucalyptus 5 FPU/gdp; 16 h	1.21	0.43
Thermomechanical non-pretreated	1.22	0.52
Thermomechanical 40 FPU/gdp; 1 h	1.19	0.54
Thermomechanical 40 FPU/gdp; 16 h	1.17	0.57

## Data Availability

The original contributions presented in this study are included in the article/Supplementary Material. Further inquiries can be directed to the corresponding author(s).

## Supplementary Materials

The following supporting information can be downloaded at: https://www.mdpi.com/article/10.3390/ma18214968/s1, Table S1. Glucan composition (%) after the enzymatic hydrolysis of non-pretreated and enzymatically pretreated pulps, analyzed by a one-way ANOVA followed by Tukey’s post hoc test at the 95% confidence level. Means not sharing a letter are significantly different, Table S2. Xylan composition (%) after the enzymatic hydrolysis of non-pretreated and enzymatically pretreated pulps, analyzed by a one-way ANOVA followed by Tukey’s post hoc test at the 95% confidence level. Means not sharing a letter are significantly different, Table S3. Total lignin composition (%) after the enzymatic hydrolysis of non- pretreated and enzymatically pretreated pulps, analyzed by a one-way ANOVA followed by Tukey’s post hoc test at the 95% confidence level. Means not sharing a letter are significantly different, Table S4. Solid recovery (%) after the enzymatic hydrolysis of non-pretreated and enzymatically pretreated pulps, analyzed by a one-way ANOVA followed by Tukey’s post hoc test at the 95% confidence level. Means not sharing a letter are significantly different, Table S5. ANOVA statistical analysis of the tensile strength (MPa) of paper produced from non-pretreated and enzymatically pretreated pulps, by a one-way ANOVA followed by Tukey’s post -hoc test at 95% confidence. Means not sharing a letter are significantly different, Table S6. ANOVA statistical analysis of the internal bonding (J/m2) of paper produced from non-pretreated and enzymatically pretreated pulps, by a one-way ANOVA followed by Tukey’s post -hoc test at 95% confidence. Means not sharing a letter are significantly different, Table S7. ANOVA statistical analysis of the air permeability (μ m/Pa·s) of paper produced from non-pretreated and enzymatically pretreated pulps, by a one-way ANOVA followed by Tukey’s post -hoc test at 95% confidence. Means not sharing a letter are significantly different, Table S8. ANOVA statistical analysis of the contact angle (°) of paper produced from non-pretreated and enzymatically pretreated pulps, by a one-way ANOVA followed by Tukey’s post -hoc test at 95% confidence. Means not sharing a letter are significantly different.
