# Supplementary material for: Advancing Sustainable Production of High-Performance Cellulose Pulps
Source: Materials (Basel). 2025 Oct 30;18(21):4968. doi: 10.3390/ma18214968 (PMC12610377; doi:10.3390/ma18214968)
Supplement: Supplementary file 1 [file materials-18-04968-s001.zip › materials-3936857-supplementary.pdf]

Supplementary Ddata

Formatted: Font color: Auto

Advancing Sustainable Production of High-Performance Cellulose Pulps

María Guadalupe Morán-Aguilar[1,*], Iván Costa-Trigo[2], Gabriela Bastida[3], André Mazega[3], Josep Duran[1], José Manuel Domínguez[4], Fabiola Vilaseca[1*]

[1] Advanced Biomaterials and Nanotechnology (BIMATEC), Department of Chemical and Agricultural Engineering, and Agrifood Technology, University of Girona, C/ M. Aurèlia Capmany, 61, 17003 Girona, Spain
[2] LNEG—National Laboratory of Energy and Geology, Bioenergy and Biorefineries Unit, Estrada do Paço do Lumiar, 22, 1649-038 Lisbon, Portugal
[3] LEPAMAP-PRODIS Research Group, Department of Chemical and Agricultural Engineering, and Agrifood Technology, University of Girona, C/Maria Aurèlia Capmany, 61, 17003 Girona, Spain
[4] Industrial Biotechnology and Environmental Engineering Group "BiotecnIA", Chemical Engineering Department, University of Vigo (Campus Ourense), 32004 Ourense, Spain

* Correspondence: lu.moag91@gmail.com; and fabiola.vilaseca@udg.edu

Table S1. Glucan composition (%) after the enzymatic hydrolysis of non-pretreated and enzymatically pretreated pulps, analyzed by a one-way ANOVA followed by Tukey's post hoc test at the 95% confidence level. Means not sharing a letter are significantly different.

Pulp	Time (h)	Enzymatic load (FPU/gdp)	Grouping letters
SP	-	-	A
SP	16	10	AB
SP	1	10	ABC
SP	1	5	ABCD
SP	16	5	BCD
SP	1	20	BCD
EP	-	-	BCDE
SP	1	40	BCDEF
SP	16	20	BCDE
EP	16	5	CDEFG
SP	16	40	DEFG
EP	16	10	EFGH
TMP	16	20	FGHI
EP	1	40	GHI
EP	1	5	HIJ
EP	1	20	HIJ
EP	1	10	IJK
EP	16	40	IJKL
TMP	16	5	JKLM
TMP	16	20	KLM
TMP	-	-	LM
TMP	1	5	M
TMP	1	10	M
TMP	16	10	M
TMP	1	40	M
TMP	1	20	M
TMP	16	-	M

SP: sulphite pulp;, EP: bleached Kraft eucalyptus pulp; TMP: thermomechanical pine pulp; -: non-pretreated pulp

Table S2. Xylan composition (%) after the enzymatic hydrolysis of non-pretreated and enzymatically pretreated pulps, analyzed by a one-way ANOVA followed by Tukey's post hoc test at the 95% confidence level. Means not sharing a letter are significantly different.

Pulp	Time (h)	Enzymatic load (FPU/gdp)	Grouping letters
TMP	16	20	A
TMP	1	5	AB
EP	-	-	ABC
TMP	16	5	ABC
TMP	1	10	ABC
TMP	-	-	ABCD
TMP	1	20	ABCD
TMP	16	10	ABCD
TMP	1	40	ABCD
EP	16	5	ABCD
EP	1	5	ABCD
TMP	16	40	ABCD
EP	1	40	ABCD
EP	16	10	ABCD
EP	1	20	BCDE
EP	1	10	BCDE
EP	16	20	CDE
EP	16	40	DEF
SP	1	5	EFG
SP	1	40	FG
SP	1	10	G
SP	1	20	G
SP	-	-	G
SP	16	40	G
SP	16	20	G
SP	16	10	G
SP	16	5	G

SP: sulphite pulp;, EP: bleached Kraft eucalyptus pulp; TMP: thermomechanical pine pulp; -: non-pretreated pulp

Table S3. Total lignin composition (%) after the enzymatic hydrolysis of non-pretreated and enzymatically pretreated pulps, analyzed by a one-way ANOVA followed by Tukey's post hoc test at the 95% confidence level. Means not sharing a letter are significantly different.

Pulp	Time (h)	Enzymatic load (FPU/gdp)	Grouping letters
TMP	16	40	A
EP	16	40	A
TMP	16	20	A
TMP	1	10	AB
TMP	16	5	AB
TMP	1	40	AB
TMP	16	10	ABC
TMP	1	5	BCD
TMP	1	20	BCD
EP	16	10	CDE
EP	16	20	DE
TMP	-	-	DE
EP	16	40	E
SP	16	20	F
SP	16	10	G
EP	1	40	H
EP	1	20	HI
EP	16	5	HI
SP	1	40	I
EP	1	10	I
EP	1	5	I
SP	1	5	I
SP	1	10	IJ
SP	1	20	IJK
SP	16	5	IJK
SP	-	-	JK
EP	-	-	K

SP: sulphite pulp;, EP: bleached Kraft eucalyptus pulp; TMP: thermomechanical pine pulp; -: non-pretreated pulp

Table S4. Solid recovery (%) after the enzymatic hydrolysis of non-pretreated and enzymatically pretreated pulps, analyzed by a one-way ANOVA followed by Tukey's post hoc test at the 95% confidence level. Means not sharing a letter are significantly different.

Pulp	Time (h)	Enzymatic load (FPU/gdp)	Grouping letters
TMP	-	-	A
EP	-	-	A
SP	-	-	A
SP	1	5	A
TMP	1	5	A
TMP	1	10	A
TMP	1	20	A
TMP	1	40	AB
SP	1	10	AB
EP	1	40	BC
SP	1	40	BCD
EP	1	20	CD
SP	1	20	CD
SP	16	5	CD
EP	1	5	CDE
TMP	16	20	CDE
TMP	16	5	CDE
EP	1	10	DE
TMP	16	10	EF
TMP	16	40	FG
SP	16	10	GH
EP	16	5	H
SP	16	20	I
EP	16	10	J
SP	16	40	J
EP	16	20	K
EP	16	40	L

SP: sulphite pulp;; EP: bleached Kraft eucalyptus pulp; TMP: thermomechanical pine pulp; -: non-pretreated pulp

Table S5. ANOVA statistical analysis of the tensile strength (MPa) of paper produced from non-pretreated and enzymatically pretreated pulps, by a one-way ANOVA followed by Tukey's post-hoc test at 95% confidence. Means not sharing a letter are significantly different.

Pulp	Time (h)	Enzymatic load (FPU/gdp)	Grouping letters
EP	1	5	A
EP	1	10	AB
TMP	1	40	B
EP	1	20	B
TMP	1	20	C
TMP	16	40	CD
TMP	16	20	CD
TMP	1	5	CDE
SP	1	10	CDE
TMP	16	5	CDE
TMP	-	-	CDE
TMP	16	10	DEF
SP	1	40	EFG
TMP	1	10	EFG
EP	-	-	EFG
SP	1	5	EFGH
SP	1	20	EFGH
EP	16	5	FGH
EP	1	40	FGHIJ
SP	16	10	GHIJ
EP	16	10	GHIJ
SP	16	20	HIJ
SP	16	40	HIJ
SP	16	5	IJ
EP	16	20	IJ
EP	16	40	J
SP	-	-	K

SP: sulphite pulp; EP: bleached Kraft eucalyptus pulp; TMP: thermomechanical pine pulp; -: non-pretreated pulp

Table S6. ANOVA statistical analysis of the internal bonding (J/m^2) of paper produced from non-pretreated and enzymatically pretreated pulps, by a one-way ANOVA followed by Tukey's post-hoc test at 95% confidence. Means not sharing a letter are significantly different.

Pulp	Time (h)	Enzymatic load (FPU/gdp)	Grouping letters
SP	16	40	A
EP	16	40	AB
TMP	16	20	AB
EP	16	20	ABC
SP	16	20	ABCD
EP	16	10	BCDE
TMP	16	10	CDEF
TMP	16	40	CDEF
TMP	-	-	DEFG
SP	16	10	DEFG
TMP	1	40	DEFGH
EP	16	5	DEFGH
SP	16	5	EFGH
EP	1	40	FGHI
TMP	16	5	FGHI
TMP	1	20	GHIJ
SP	1	10	GHIJ
TMP	1	10	GHIJ
EP	1	20	GHIJ
SP	1	5	GHIJ
EP	1	10	GHIJ
EP	1	5	GHIJ
SP	-	-	GHIJ
SP	1	40	GHIJ
SP	1	20	HIJ
TMP	1	5	IJ
EP	-	-	J

SP: sulphite pulp; EP: bleached Kraft eucalyptus pulp; TMP: thermomechanical pine pulp; -: non-pretreated pulp

Table S7. ANOVA statistical analysis of the air permeability (μm/Pa·s) of paper produced from non-pretreated and enzymatically pretreated pulps, by a one-way ANOVA followed by Tukey's post-hoc test at 95% confidence. Means not sharing a letter are significantly different.

Pulp	Time (h)	Enzymatic load (FPU/gdp)	Grouping letters
SP	-	-	A
EP	1	40	AB
TMP	16	10	AB
EP	1	20	ABC
TMP	1	20	ABC
EP	1	10	ABCD
TMP	1	5	ABCDE
TMP	1	40	ABCDE
TMP	1	10	ABCDE
EP	-	-	ABCDE
SP	1	5	ABCDE
EP	1	5	ABCDEF
SP	1	20	ABCDEF
TMP	16	5	ABCDEF
TMP	16	20	CDEFG
EP	16	5	DEFGH
SP	1	10	EFGH
SP	16	5	EFGH
TMP	-	-	FGHI
EP	16	10	FGHI
SP	1	40	GHI
SP	16	10	GHIJ
TMP	16	40	HIJ
EP	16	20	IJK
SP	16	20	JK
EP	16	40	JK
SP	16	40	K

SP: sulphite pulp;, EP: bleached Kraft eucalyptus pulp; TMP: thermomechanical pine pulp; -: non-pretreated pulp

Table S8. ANOVA statistical analysis of the contact angle (°) of paper produced from non-pretreated and enzymatically pretreated pulps, by a one-way ANOVA followed by Tukey's post-hoc test at 95% confidence. Means not sharing a letter are significantly different.

Pulp	Time (h)	Enzymatic load (FPU/gdp)	Grouping letters
TMP	1	20	A
TMP	1	10	AB
TMP	1	40	ABC
SP	16	40	ABCD
SP	16	20	ABCDE
EP	1	20	ABCDE
TMP	1	5	ABCDEF
SP	1	40	ABCDEFG
TMP	16	40	ABCDEFG
TMP	16	10	ABCDEFG
TMP	16	20	BCDEFGH
EP	1	40	BCDEFGHI
SP	16	10	CDEFGHI
TMP	16	5	CDEFGHI
EP	1	10	DEFGHI
SP	16	5	DEFGHI
EP	16	5	DEFGHI
EP	1	5	EFGHI
EP	16	10	FGHI
SP	1	20	GHI
EP	16	20	HI
EP	16	40	HI
SP	1	10	IJ
SP	1	5	IJ
TMP	-	-	IJ
EP	-	-	JK
SP	-	-	K

SP: sulphite pulp; EP: bleached Kraft eucalyptus pulp; TMP: thermomechanical pine pulp; -: non-pretreated pulp
